# Impact of the COVID-19 pandemic on liver transplant waitlist outcome in France

**DOI:** 10.1038/s41598-023-32680-8

**Published:** 2023-06-08

**Authors:** Camille Legeai, Corinne Antoine, Carine Jasseron, François Kerbaul, Jérôme Dumortier

**Affiliations:** 1 Organ and Tissue Procurement and Transplantation Department, Agence de la Biomédecine, 1, Avenue du Stade de France, 93212 Saint-Denis la Plaine Cedex, France; 2 Hospices Civils de Lyon, Hôpital Edouard Herriot, Unité de Transplantation Hépatique et Université Claude Bernard Lyon 1, Lyon, France

**Keywords:** Epidemiology, Liver diseases

## Abstract

The objective of this study was to investigate the impact of the COVID-19 pandemic on the outcome of patients on the liver transplantation (LT) waitlist in 2020 in France, in particular, the incidence of deaths and delisting for worsening condition, depending on the allocation score component. The 2020 cohort of patients on the waiting list was compared with the 2018/2019 cohorts. 2020 saw fewer LTs than in either 2019 or 2018 (1128, 1356, and 1325, respectively), together with fewer actual brain dead donors (1355, 1729, and 1743). In 2020, deaths or delisting for worsening condition increased significantly versus 2018/2019 (subdistribution hazard ratio 1.4, 95% confidence interval [CI] 1.2–1.7), after adjustment for age, place of care, diabetes, blood type, and score component, although COVID-19-related mortality was low. This increased risk mainly concerned patients with hepatocellular carcinoma (1.52, 95% CI 1.22–1.90), with 650 MELD exception points (2.19, 95% CI 1.08–4.43), and especially those without HCC and MELD scores from 25 to 30 (3.36 [95% CI 1.82–6.18]). In conclusion, by significantly decreasing LT activity in 2020, the COVID-19 pandemic increased the number of waitlist deaths and delisting for worsening condition, and significantly more for particular components of the score, including intermediate severity cirrhosis.

## Introduction

Coronavirus disease-2019 (COVID-19), caused by severe acute respiratory syndrome coronavirus-2 (SARS-CoV-2), is an ongoing global pandemic of major concern. In France, it began in early 2020 and has profoundly affected the French health care system, and indeed these systems worldwide. The novelty of the disease initially created tremendous uncertainty about its potential impact on solid organ transplant candidates and recipients, especially due to the expected high disease incidence and the increased risk of adverse outcomes and deaths among these recipients. The first large nationwide report confirmed these fears^1^. These uncertainties also led transplant centers to act cautiously in admitting, operating on, and immunosuppressing patients during this pandemic. In addition, liver transplantation (LT), patients with cirrhosis, particularly decompensated cirrhosis, were rapidly recognized as a population at high risk of a poor prognosis if infected by COVID-19^1,2^. These uncertainties, which evolved into preliminary results, and, together with considerable increases in COVID-19–related hospitalizations and deaths, may have strongly affected organ procurement activity in general and LT in particular.

The aims of the French national study presented here were to: (1) compare the incidence of death on the waiting list and of delisting due to patients' worsening condition on the liver waitlist in 2020 with those who were actively registered during the same periods in 2018 and 2019 and (2) measure whether the observed difference varied according to the indication for transplantation.

## Results

### Global description of the cohort

**Figure 1:**
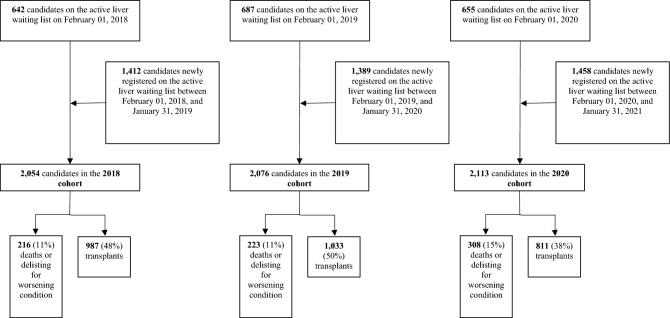
Flow diagram of inclusion and follow-up.

The active liver waiting list on February 01, 2018, included 642 patients, 687 on February 01, 2019, and 655 on February 01, 2020 (Fig. 1). The list added 1412 newly active patients between February 2018 and January 2019, 1389 between February 2019 and January 2020, and 1458 between February 2020 and January 2021.

After an average follow-up of 4.8 months (3.7 months for newly actively registered patients and 7.1 months for patients on the active liver waiting list on February 01), 2831 (45.3%) patients had been transplanted, 444 (7.1%) had died, and 303 (4.9%) had been delisted for worsening condition.

### COVID-19

In the 2020 cohort, 82 (4.2%) patients were infected with SARS-Cov2; 19 (23%) died or were delisted, and these percentages did not differ significantly between indications for transplantation. These 19 deaths accounted for 0.9% (19/2113) of the 2020 cohort and 6% (19/308) of the total of deaths and delistings for worsening condition in the 2020 cohort.

### Baseline candidate characteristics

Overall, at inclusion, the candidates' mean age was 56.5 years (SD: 10.6 years), 21.4% were aged 65 years or over, and 25.5% were female. When waitlisted, 28.3% had diabetes and 25.4% had a BMI higher than 30 kg/m^2^. At inclusion in the study, 22% were hospitalized, 41.9% of them in an intensive care unit.

HCC was the indication for transplant for 44.7% of the patients and cirrhosis for 38.4%. Among the latter, 24% had a MELD score < 15, 23% between 15 and 20, 17% between 20 and 25, 13% between 25 and 30, 9% between 30 and 35, and 14% between 35 and 40. Another 9.2% of candidates were allocated additional score points due to specific symptoms or diseases: 4.5% were in the 800-MEP group and 4.7% in the 650-MEP group.

Table 1 summarizes the candidate characteristics according to whether they were in the 2020 or the 2018/2019 cohorts. No significant difference was observed between the 2020 and the 2018/2019 cohorts overall, or when considering only the newly actively registered patients. The one exception was that the transplant indication of cirrhosis tended to be more frequent in 2020 newly actively registered patients (45.1 vs. 42.5%,* p* = 0.10).

**Table 1:** Candidates' characteristics at baseline by inclusion period.

		N	% missing data	2018/2019 cohorts^†^ (N = 4130)	2020 cohort^‡^ (N = 2113)	*p*
Male		4652	0	3093 (74.9)	1559 (73.8)	0.34
Age at inclusion		6243	0	56.4 (10.6)	56.6 (10.6)	0.48
Age at inclusion	< 40 years	535	0	355 (8.6)	180 (8.5)	0.88
	40–50 years	811		542 (13.1)	269 (12.7)	
	50–55 years	850		563 (13.6)	287 (13.6)	
	55–60 years	1209		809 (19.6)	400 (18.9)	
	60–65 years	1500		972 (23.5)	528 (25)	
	≥ 65 years	1338		889 (21.5)	449 (21.2)	
BMI at registration	Underweight or normal (< 25 kg/m^2^)	2568	0	1710 (41.4)	858 (40.6)	0.83
	Overweight (25–30 kg/m^2^)	2089		1375 (33.3)	714 (33.8)	
	Obese (> 30 kg/m^2^)	1586		1045 (25.3)	541 (25.6)	
Blood group	A	2715	0	1791 (43.4)	924 (43.7)	0.54
	B	655		434 (10.5)	221 (10.5)	
	AB	187		133 (3.2)	54 (2.6)	
	O	2686		1772 (42.9)	914 (43.3)	
Diabetes at registration		1738	1.7	1147 (28.3)	591 (28.4)	0.89
Place of care	Home	4884	0.1	3240 (78.5)	1644 (77.9)	0.77
	Hospital	786		518 (12.6)	268 (12.7)	
	Intensive Care Unit	566		367 (8.9)	199 (9.4)	
Transplant indication	Cirrhosis MELD < 25	1522	0.2	1002 (24.3)	520 (24.6)	0.15
	Cirrhosis and MELD [25–30]	308		201 (4.9)	107 (5.1)	
	Cirrhosis and MELD [30–35]	224		141 (3.4)	83 (3.9)	
	Cirrhosis and MELD ≥ 35	330		204 (4.9)	126 (6)	
	Hepatocellular carcinoma	2791		1862 (45.2)	929 (44)	
	650-MEP^§^	296		183 (4.4)	113 (5.4)	
	800-MEP^¶^	280		195 (4.7)	85 (4)	
	Other	482		335 (8.1)	147 (7)	

^†^2018–19 cohorts: candidates active on the waiting list on February 1, 2018, patients actively registered on the waiting list for the first time between February 2018 and January 2019, candidates active on the waiting list on February 1, 2019 and patients actively registered on the waiting list for the first time between February 2019 and January 2020.

^‡^2020 cohort: candidates active on the waiting list on February 1, 2020 and patients actively registered on the waiting list for the first time between February 2020 and January 2021.

^§^650-MEP: 650 MELD exception points were given to candidates with chronic encephalopathy and MELD < 15, refractory ascites and MELD < 15 or untreatable HCC.

^¶^800-MEP: 800 MELD exception points were given to candidates with recurrent gastrointestinal bleeding and MELD < 15, hepatopulmonary syndrome, portopulmonary hypertension, HIV infection, refractory pruritus, recurrent bacterial cholangitis, Budd-Chiari syndrome, hereditary hemorrhagic telangiectasia, polycystic liver disease, familial amyloid polyneuropathy, cholangiocarcinoma, hepatic metastases in gastrointestinal endocrine tumors, hepatic epithelioid hemangioendothelioma, primary sclerosing cholangitis, or primary biliary cirrhosis.

### Cumulative incidence of transplant and death or delisting in the 2020 versus the 2018/2019 cohorts

**Figure 2:**
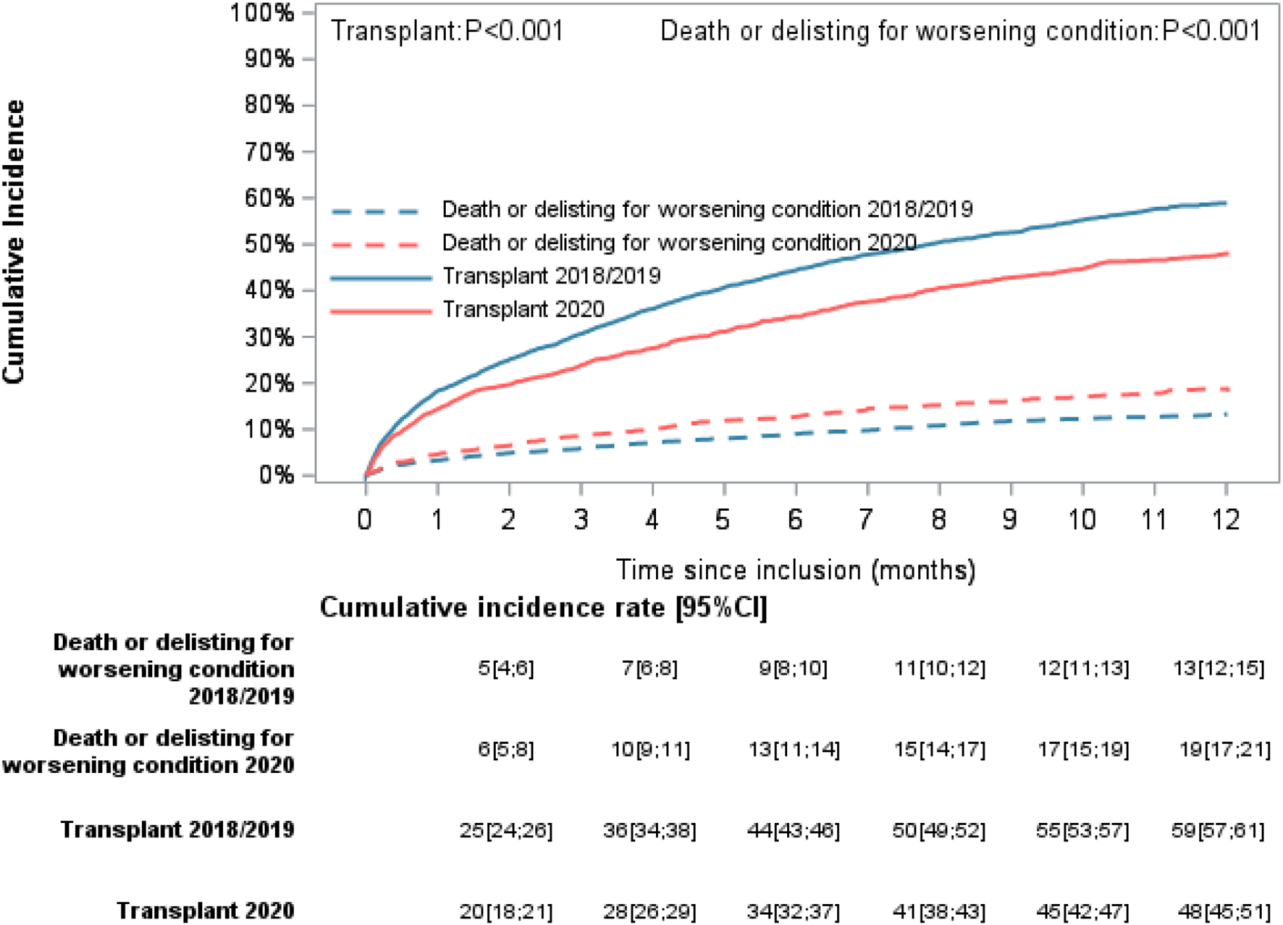
Cumulative incidence of transplant and death or delisting for worsening condition by inclusion period.

Figure 2 shows the cumulative incidence functions of transplant and separately of death or delisting according to the inclusion period. From the first month post-inclusion, the cumulative incidence of transplant was significantly lower in the 2020 cohort. Six months post-inclusion, the cumulative transplant incidence was equal to 34% (95%CI 32–37) in the 2020 cohort versus 44% (95% CI 43–46) in the combined 2018/2019 cohorts. The cumulative incidence of death or delisting was significantly higher in the 2020 cohort at 3 months after inclusion. Six months after inclusion, the cumulative incidence of death or delisting was equal to 13% (95% CI 11–14) in the 2020 cohort versus 9% (95% CI 8–10) in the 2018/2019 cohorts.

We observed a significant interaction between inclusion period and the transplant indication group, for both the cumulative transplant incidence (*p* for interaction < 0.01) and the cumulative incidence of death or delisting (*p* for interaction = 0.04).

**Figure 3:**
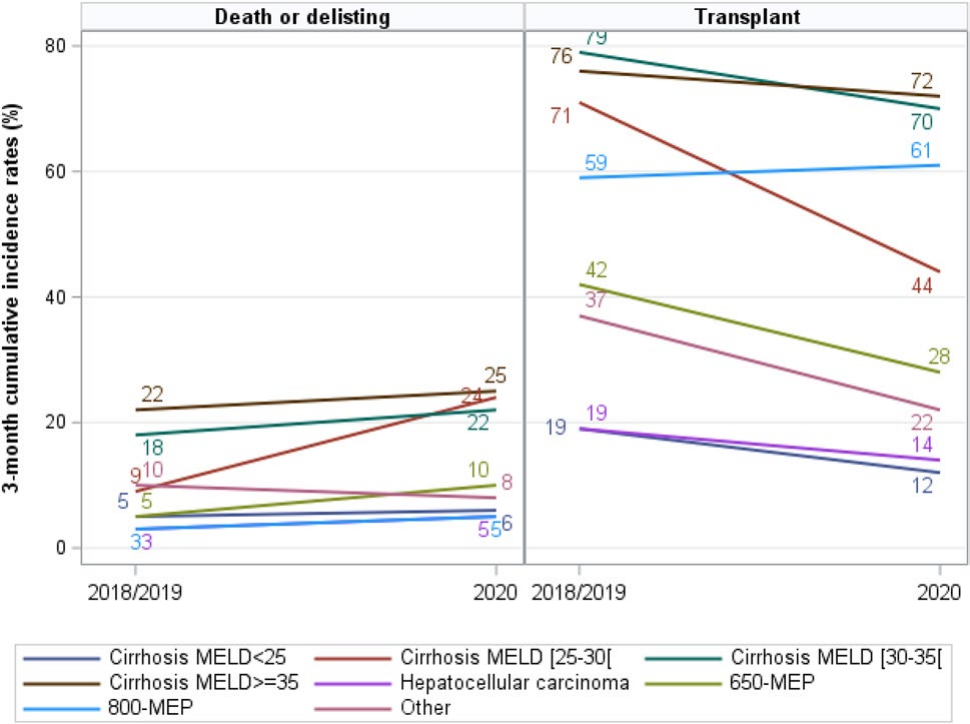
3-month cumulative incidence of death or delisting for worsening condition and transplant by inclusion period and transplant indication group.

The 6-month cumulative transplantation incidence decreased by 31.2% (95% CI 26.9–34.7) among patients with cirrhosis and MELD scores of 25–30 between the 2018/2019 and the 2020 cohorts, although the decrease for the other transplant indication groups was only 8.9% (95% CI 8.3–9.4) (Fig. 3). The 3-month cumulative incidence of death or delisting increased by 15.4% (95% CI 10.8–19.8) for patients with cirrhosis and MELD scores of 25–30 between the 2018/2019 and the 2020 cohorts, but only 1.9% (95% CI 1.5–2.5) in the other transplant indication groups (Fig. 3). The 6-month cumulative incidence of death or delisting increased even more between the 2018/2019 and 2020 cohorts for patients with cirrhosis and MELD scores of 25–30 (16.9%, 95% CI 12.0–21.4). An increase in the 6-month cumulative incidence of death or delisting also became apparent for patients in both the 650-MEP (9.4%, 95% CI 6.1–12.8) and the 800-MEP (3.4%, 95% CI 1.2–7.0) groups, as well as for those with HCC (3.6%, 95% CI 2.8–4.6). There remained no significant difference in the other groups (1.2%, 95% CI 0.6–2.1).

### Impact on the 2020 cohort by indication group

In multivariable analysis, the factors significantly associated with a higher incidence of death or delisting were age of 50 years and over, diabetes, blood group other than A, hospitalization at inclusion, “cirrhotic patients with MELD ≥ 35 as transplant indication group, and 2020 as the inclusion period. The subdistribution hazard of death or delisting was 44% (95% CI 24–67) higher and the subdistribution hazard of transplantation 30% (95% CI 24–36) lower for patients in the 2020 cohort compared to those in the 2018/2019 cohorts. After adjustment for age, diabetes, blood group, and place of care, the interaction between the inclusion period and the transplant indication group was still significant for the cumulative incidence of death or delisting (*p* = 0.035) or of transplantation (*p* = 0.016).

**Figure 4:**
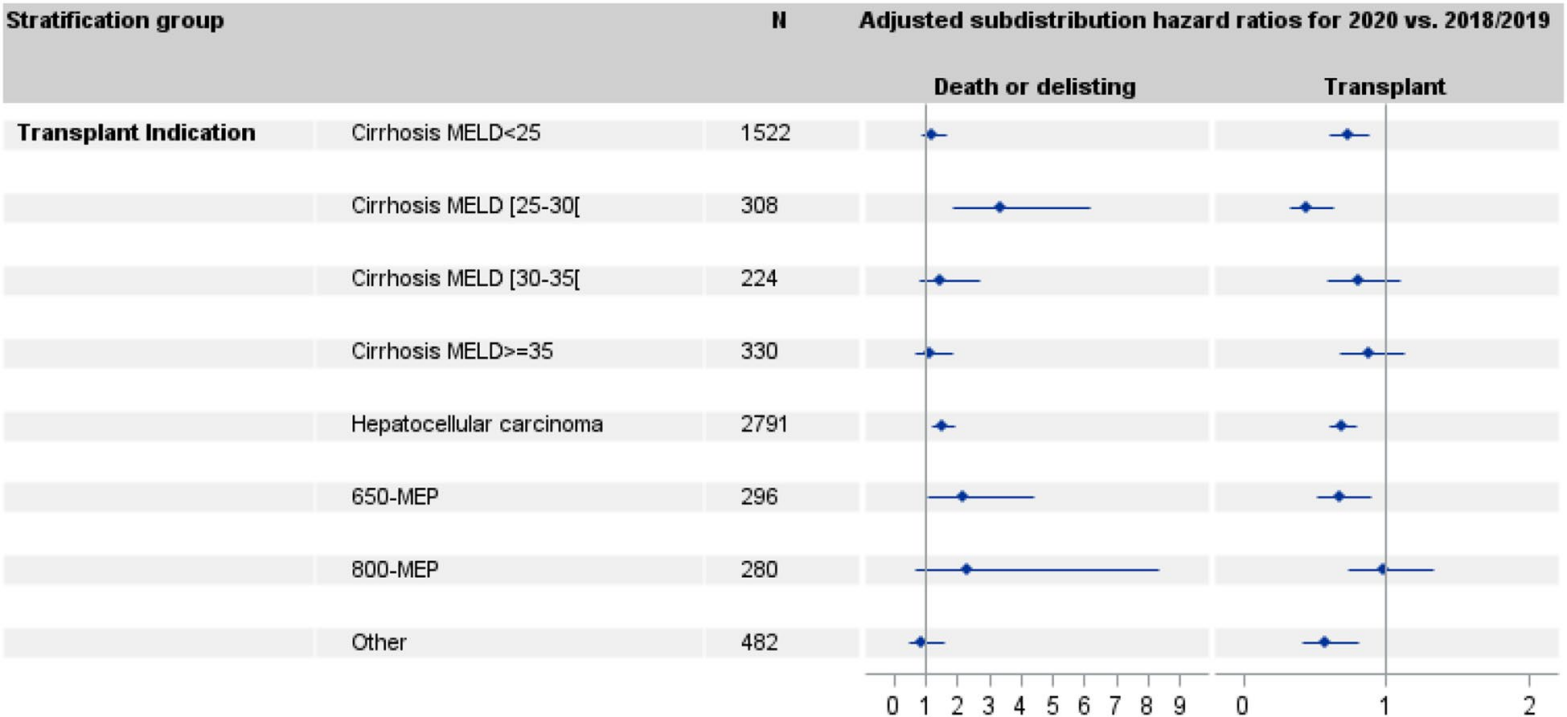
Adjusted subdistribution hazard ratios for transplant and death or delisting for worsening condition for the 2020 versus 2018/19 cohorts for each transplant indication group.

Multivariable analyses were conducted separately in each transplant indication group. Figure 4 reports the adjusted subdistribution hazard ratios for death or delisting and for transplant for the 2020 versus the 2018/2019 cohorts and confirms that the adjusted effect of the year of inclusion (2020 vs. 2018/2019) differed by the transplant indication group. For the patients with cirrhosis and a MELD score of 25–30, the subdistribution hazard of death or delisting was 3.4 (95% CI 1.8–6.2) times higher and that of transplant 2.2 (95% CI 1.6–3.1) times lower for the 2020 than the 2018/2019 cohorts. In patients with HCC, the subdistribution hazard of death or delisting was 1.5 (95% CI 1.2–1.9) times higher and that of transplant 1.4 (95% CI 1.3–1.7) times lower for the 2020 than the 2018/2019 cohorts. Patients in the 650-MEP group had a subdistribution hazard of death or delisting 2.2 (95% CI 1.1–4.4) times higher and a subdistribution hazard of transplant 1.5 (95% CI 1.1–1.2) times lower in the 2020 than in the 2018/2019 cohorts. On the other hand, the inclusion period was not significantly associated with a change in the subdistribution hazard of death or delisting or in that of transplant in cirrhotic patients with MELD scores ≥ 30 and in patients in the 800-MEP group. In the final two categories of patients (cirrhotic patients with MELD scores < 25 and other transplant indications), the year of inclusion was associated with a decrease in the subdistribution hazard of transplant but we observed no significant change in that for death or delisting.

In the sensitivity analysis, when deaths and delisting that occurred in patients infected with SARS-Cov2 were censored, results did not change. Cirrhotic patients with MELD scores of 25–30, patients with HCC, and patients in the 650-MEP group were still the categories of patients particularly affected in 2020 with a significant decrease in the subdistribution hazard of transplant and a significant increase in the subdistribution hazard of deaths or delisting.

## Discussion

This study compared the incidence of death on the waiting list or delisting for worsening condition of the condition of patients on the active liver waiting list in 2020 to those who were actively registered in 2018 and 2019. The subdistribution hazard of death or delisting was 44% higher and that of transplant 30% lower in 2020. Only 6% of the total deaths or delistings for worsening condition in the 2020 cohort occurred in patients infected with SARS-Cov2. The deleterious effect of the 2020 cohort on the subdistribution hazard of death or delisting was particularly important in cirrhotic patients with MELD scores of 25–30, while no significant effect was observed in cirrhotic patients with MELD scores < 25 or ≥ 30. Access to transplant also decreased in patients in the 650-MEP group and those with HCC, associated with an increase in the incidence of death or delisting.

The first relevant result from our study is the low COVID-19-related mortality among waitlisted patients (0.9% of the 2020 cohort). Nevertheless, it has been previously suggested that patients with chronic liver disease, especially cirrhosis, and even more decompensated cirrhosis and/or alcohol-related liver disease, are at significant risk of adverse COVID-19 outcomes. In France, alcohol-related liver disease is the most frequent underlying liver disease in patients listed for LT. A recent meta-analysis including 63 English-language primary research articles assessed the reported clinical outcomes of patients with cirrhosis who develop COVID-19 infection^3^. Meta-analysis of cohort studies reporting a noncirrhotic comparator yielded a pooled mortality odds ratio (OR) of 2.48 (95% CI 2.02–3.04), and analysis of a subgroup of studies reporting ORs for mortality in hospitalized patients, adjusted for significant confounders, found a pooled adjusted OR of 1.81 (95% CI 1.36–2.42). Data about cirrhotic patients with decompensated cirrhosis, based on either the Child-Turcotte-Pugh classification or the MELD score, are scarce. The first available large study, by Moon et al.^4^ included 103 patients with cirrhosis and 49 with noncirrhotic chronic liver disease. Death occurred in 12.2% of patients without cirrhosis, 24% of patients with Child-Turcotte-Pugh class A cirrhosis, 43% of patients with Child-Turcotte-Pugh class B cirrhosis, and 63.0% of patients with Child-Turcotte-Pugh class C cirrhosis. The cause of death in patients with cirrhosis was reported as COVID-19 lung disease in 78.7%, and liver-related in only 12.2%. The multicenter study by Bajaj et al. compared 37 inpatients with cirrhosis and COVID-19 to age/gender-matched patients with COVID-19 alone (n = 108) or cirrhosis alone (n = 127)^5^. Patients with both cirrhosis and COVID-19 had mortality similar to that of patients with cirrhosis alone, but higher than patients with COVID-19 alone. In addition, studies including patients with alcohol-related cirrhosis have suggested increased mortality in these patients as in other patients with cirrhosis^6–8^. Of note, patients with alcohol-related liver disease often had associated comorbidities such as obesity, diabetes mellitus, and chronic kidney disease, which also increase the risk for complications in COVID-19.

From February to November 2020, the European Liver and Intestine Transplantation Association (ELITA) conducted an internet-based survey among transplant centers affiliated with the European Liver Transplant Registry (ELTR) and showed that LT candidates are at high risk of a severe course of COVID-19. The survey included 113 cases of COVID-19; thirty-seven patients (32.7%) died, with respiratory failure being the major cause (33/37). MELD score was significantly associated with mortality^9^. Finally, Mallet et al.^10^ explored the outcomes of all adult inpatients with COVID-19 in France, in 2020. The authors computed adjusted ORs to measure the associations between chronic liver disease, alcohol use disorders, mechanical ventilation, and 30-day in-hospital mortality. The sample comprised 259,110 patients, including 15,476 (6.0%) and 10,006 (3.9%) with chronic liver disease and alcohol use disorders, respectively. Patients with alcohol use disorders, decompensated cirrhosis, or primary liver cancer were at higher risk of COVID-19-related mortality but less likely to receive mechanical ventilation. These results are highly relevant to our study of patients waitlisted for LT in France.

The number of LTs performed during the first year of the COVID-19 pandemic fell from previous levels^11^. This 16.6% reduction was directly related to a 17.0% reduction in the number of livers retrieved and, more largely, a 21.0% in the number of donors. Liver procurement efficiency did not decrease, as livers were procured from 77% of the actual deceased donors. The number of new waitlisted patients was stable. Kwapisz et al. compared the effects of the COVID-19 pandemic on solid organ transplantation during 2020 in Poland with countries in Western Europe, North America, and Asia^12^. In Poland, for the first 10 months of the pandemic (from March to December 2020), the monthly number of deceased organ donors decreased by 42% from its prepandemic value (42 vs. 24 per month). As a result, the number of all solid organ transplants decreased by 36%, and the number of deceased donors LT by 63%.

Although Spain has been the world leader in organ donation and transplantation in recent years, it was one of the European countries most affected by COVID-19, which initially reduced the mean number of donors dramatically—from 7.2 to 1.2 per day and the mean number of transplants from 16.1 to 2.1 per day^13^. Interestingly, in some centers, or regions, or countries, living-donor LT activity did not fall significantly, even at the epidemic's peak. This was the case, for example, in South Korea, because of the strict screening and tracing policy based on the experience of the previous Middle Eastern Respiratory Syndrome Coronavirus (MERS-CoV) infection^14^. It can be hypothesized that organ procurement decreased significantly because of the intensive mobilization of medicine resources for the care of COVID-19 patients. The fear of transmission of SARS-CoV2 probably did not have a significant impact^15^.

Our results can also be compared with the US Scientific Registry of Transplant Recipients data. Strauss et al. compared observed LT waitlist registrations, waitlist mortality, deceased donor LT, and living donor LTs from March 15 through August 31, 2020, with expected values based on historical trends from January 2016 through January 2020, stratified by statewide COVID-19 incidence^16^. Overall, from March 15 through April 30, 2020, there were 11% fewer new listings than expected, 49% fewer living donor LTs, and 9% fewer deceased donor LTs. In May 2020, there were 21% fewer new listings, 42% fewer living donor LTs, and 13% more deceased donor LTs. Centers in states with the highest COVID-19 incidence rates from March 15 to April 30, 2020, had 59% more waitlist deaths and 34% fewer deceased donor LTs. In France, we also observed major geographical disparities in COVID-19 incidence and LT activity (data not shown).

Our study provides comprehensive, original information on the impact of the liver graft shortage on access to LT, and therefore of the mortality risk of patients on the waiting list, according to the indication for transplantation. It must be put into perspective in view of the 3 different scenarios observed during the pandemic among centers worldwide: complete shutdown of activity, limitation of transplant activity favoring a “sickest-first” approach, or continuation of routine transplant activity. In France, the scenario observed, more than deliberately chosen, was the "sickest first" one, for LT. This differed from kidney transplantation, for which activity decreased drastically for 3 months. The reduction of activity did not induce a significant risk for mortality for “very sick” patients, estimated by MELD score (i.e., MELD score > 30), or under the MELD “800 points” exception. The reduction of LT access for the “least sick” patients (MELD score < 25) also did not induce excess mortality. On the other hand, the patients with MELD scores of 25–30 and those under the MELD “650 points” exception were significantly more affected. Although the observed impact on HCC patients appeared limited, longer-term reevaluation will be required as their prognosis decreases progressively with time, with a progressive risk of delisting.

Our study should be interpreted in the context of its limitations. First, some patients may have changed transplant indication group while on the waiting list. However, the fact that the increase in the subdistribution hazard of death or delisting in 2020, notably in cirrhotic patients with MELD 25–30, was associated with a decrease in the subdistribution hazard of transplant, shows that these group changes only concerned a small number of patients. Second, due to an initial lack of SARS-Cov2 testing, there were likely more deaths on the waiting list due to COVID-19 in 2020 than recorded in the CRISTAL registry and reported here. However, the disparity in the distribution of the 2020 excess of deaths or delistings between transplant groups and the parallel decrease in access to transplantation in the groups where the excess of deaths or delistings was more pronounced suggest that the impact of COVID-19-related mortality among waitlisted patients was nevertheless limited compared to that of the decrease in access to transplantation. Finally, the specificity of the French liver allocation system make our results difficult to compare with what has been observed in other countries during the pandemic.

The study will be continued to refine the results and assess the longer-term impact of the COVID-19 health crisis together with the impact of SARS-Cov2 vaccination. This analysis has nevertheless already made it possible to highlight imperfections in the graft allocation system, in situations of graft shortage such as that caused by this situation. Work is underway to determine how to improve the French liver allocation system. Other countries chose to modify their allocation system during the COVID-19 pandemic, with, for example, the prioritization of waitlist recipients with the highest need in the United Kingdom^17^. This adaptation must certainly be discussed to maintain equity in the risk of death or delisting among the different indications for LT.

## Material and methods

### Study population and data sources

Our data come from the French registry, CRISTAL, a national database that began in 1996 and is administered by the Agence de la biomedicine. It prospectively collects data about all organ transplant candidates and recipients in France, together with their outcomes. The demographic, clinical, and laboratory data of candidates for LT are collected by transplantation teams at waitlisting and every three months thereafter until transplantation. Candidates' deaths and delistings are reported prospectively.

Additional data concerning COVID-19 have been collected since the start of the epidemic in France from both candidates and recipients. The diagnostic criteria for COVID-19 were as follows: (1) evidence of SARS-CoV-2 infection on reverse transcriptase-polymerase chain reaction (RT-PCR) testing performed on nasopharyngeal swab specimens, or (2) presence of typical respiratory symptoms accompanied by evocative pulmonary lesions on low-dose chest computed tomography, even when RT-PCR yielded negative results, or (3) clinical diagnosis only.

Data accuracy is verified by CRISTAL research assistants. The study was conducted in accordance with French law. Because French legislation defines research studies based on the CRISTAL registry as part of transplant outcome assessment, they do not require ethics committee review or approval. Informed consent was obtained from all subjects and/or their legal guardian(s).

Three cohorts were analyzed. Each cohort included patients who were registered on the French national waiting list for single-organ LT. The 2020 cohort included candidates who were active on the waiting list on February 1, 2020 (N = 655), and those who were actively registered on the waiting list for the first time between February 2020 and January 2021 (N = 1458); the 2019 cohort included candidates who were active on the waiting list on February 1, 2019 (N = 687), and patients who were actively registered on the waiting list for the first time between February 2019 and January 2020 (N = 1389); the 2018 cohort included candidates who were active on the waiting list on February 1, 2018 (N = 642), and patients who were actively registered on it for the first time between February 2018 and January 2019 (N = 1412).

Among them, those younger than 18 at the time of waitlisting (N = 413) were excluded, as were those with an ongoing high-urgency priority status at inclusion (N = 400, including 193 with an acute liver failure secondary to fulminant hepatitis), and those waiting for combined organ (N = 462) or potential living donor transplantation (N = 208).

For each cohort, date of inclusion was: (1) February 1, for candidates who were active on the waiting list on that date, or (2) the day of the first active registration for candidates who were actively registered on the waiting list for the first time between February and January of the following year. The endpoint was death on the waiting list or delisting for worsening condition (henceforth, unless specifically noted, delisting means delisting for worsening condition). Data were censored on January 31, 2021, for the 2020 cohort, January 31, 2020, for the 2019 cohort, and January 31, 2019, for the 2018 cohort, or at the time of delisting for a cause other than worsening condition, whichever came first.

This study considered eight groups corresponding to the point assignment categories of the French allocation system^18^. In this system, besides superurgent LT, the patients with the most points are transplanted first. In patients with decompensated cirrhosis, points are awarded according to the Model for End-Stage Liver Disease (MELD) score. Patients with hepatocellular carcinoma (HCC) are prioritized according to their MELD score but also their tumor status, response to alternative therapy, and waiting time. Besides decompensated cirrhosis and HCC, several MELD exceptions are also possible. A maximum of either 650 or 800 points can be granted immediately or within 3, 6 or 12 months, according to the opinion of 2 experts, for patients with fewer than 15 MELD points. Thus, 800 additional points can be given to patients with the following conditions: recurrent gastrointestinal bleeding and MELD < 15, hepatopulmonary syndrome, portopulmonary hypertension, HIV infection, refractory pruritus, recurrent bacterial cholangitis, Budd-Chiari syndrome, hereditary hemorrhagic telangiectasia, polycystic liver disease, familial amyloid polyneuropathy, cholangiocarcinoma, hepatic metastases in gastrointestinal endocrine tumors, hepatic epithelioid hemangioendothelioma, primary sclerosing cholangitis, and primary biliary cirrhosis; 650 additional points can be given to patients with chronic severe encephalopathy, refractory ascites, or untreatable HCC. In this study, the cirrhosis indication was divided into four groups according to the MELD score at inclusion: < 25, 25–30, 30–35, and ≥ 35. Another group comprised candidates with HCC. Candidates with 800 MELD exception points were in the 800-MEP group and those with 650 MELD exception points in the 650-MEP group, whatever the initial liver disease. The eighth group included all other candidates (Supplementary Tables S1and S2).

### Statistical analysis

Continuous variables are reported as means (standard deviation, SD) and categorical variables as frequencies (percentages). Differences between groups were assessed with the Chi-2 and t-tests.

The cumulative incidence functions of transplant and death or delisting were estimated and then compared between groups by using Gray’s test. Factors associated with the cumulative incidence of waitlist mortality or delisting were determined with a multivariable Fine-Gray subdistribution hazard regression model with LT as a competing event. Data were censored at the end of follow-up (January 31, 2021, for the 2020 cohort, and similarly for the 2019 and 2018 cohorts) or at the time of delisting for a cause other than worsening condition, whichever came first. All potential confounders were introduced into the multivariable analysis and then selected by a stepwise backward procedure. An interaction test was performed on the multivariable model obtained to assess whether the effect of the 2020 cohort on the cumulative incidence of mortality on the waiting list or of delisting was significantly different in transplant indication subgroups. The adjusted effect of the 2020 cohort was then assessed separately in the eight transplant indication subgroups. To verify that the effect of the 2020 cohort was not solely the result of excess mortality due to COVID-19, a sensitivity analysis was conducted by censoring deaths or delistings that occurred in patients infected with SARS-Cov2. All tests were two-sided and *p* < 0.05 was considered statistically significant. Analyses were performed with SAS Enterprise Guide 7.1 (SAS Institute, Inc).

## Supplementary Information

Supplementary Tables.

## Data Availability

Data available on request from the corresponding author.

## Abbreviations

CI: Confidence interval
HCC: Hepatocellular carcinoma
LT: Liver transplantation
MELD: Model for end stage liver disease
SD: Standard deviation
