# Supplementary material for: Impact of the COVID-19 pandemic on liver transplant waitlist outcome in France
Source: Sci Rep. 2023 Jun 8;13:9308. doi: 10.1038/s41598-023-32680-8 (PMC10248328; doi:10.1038/s41598-023-32680-8)
Supplement: Supplementary file 1 — Supplementary Tables. [file 41598_2023_32680_MOESM1_ESM.pdf]

Supplementary Table S1 : Time on the waiting list at inclusion according to transplant indication group in candidates on the active waiting list on February 01, 2018 or 2019 or 2020

	Cirrhosis MELD<25 (n, %)	Cirrhosis and MELD [25-30[(n, %)	Cirrhosis and MELD [30-35[(n, %)	Cirrhosis and MELD≥35 (n, %)	Hepatocellular carcinoma (n, %)	650-MEP (n, %)	800-MEP (n, %)	Other (n, %)
**Time on the waiting list at inclusion (months)**								
**<3**	122<br>25,31%	35<br>72,92%	13<br>100%	3<br>60%	232<br>22,24%	40<br>21,86%	52<br>41,27%	24<br>28,57%
**3-6**	75<br>15,56%	2<br>4,17%	0<br>0%	2<br>40%	188<br>18,02%	57<br>31,15%	34<br>26,98%	14<br>16,67%
**6-12**	105<br>21,78%	5<br>10,42%	0<br>0%	0<br>0%	340<br>32,60%	61<br>33,33%	31<br>24,60%	19<br>22,62%
**>=12**	180<br>37,34%	6<br>12,50%	0<br>0%	0<br>0%	283<br>27,13%	25<br>13,66%	9<br>7,14%	27<br>32,14%
**Total**	**482**	**48**	**13**	**5**	**1043**	**183**	**126**	**84**

Supplementary Table S2 : Time on the waiting list at inclusion according to transplant indication group in candidates newly registered between February 2018 and January 2021

	Cirrhosis MELD<25 (n, %)	Cirrhosis and MELD [25-30[(n, %)	Cirrhosis and MELD [30-35[(n, %)	Cirrhosis and MELD≥35 (n, %)	Hepatocellular carcinoma (n, %)	650-MEP (n, %)	800-MEP (n, %)	Other (n, %)
**Time on the waiting list at inclusion (months)**								
**<3**	971<br>93,37%	251<br>96,54%	206<br>97,63%	324<br>99,69%	1438<br>82,27%	79<br>69,91%	113<br>73,38%	382<br>95,98%
**3-6**	45<br>4,33%	6<br>2,31%	4<br>1,90%	0<br>0%	165<br>9,44%	26<br>23,01%	24<br>15,58%	8<br>2,01%
**6-12**	16<br>1,54%	2<br>0,77%	1<br>0,47%	1<br>0,31%	82<br>4,69%	7<br>6,19%	12<br>7,79%	5<br>1,26%
**>=12**	8<br>0,77%	1<br>0,38%	0<br>0%	0<br>0%	63<br>3,60%	1<br>0,88%	5<br>3,25%	3<br>0,75%
**Total**	**1040**	**260**	**211**	**325**	**1748**	**113**	**154**	**398**
